# An Integrated Analysis of Informal Caregiving and Psychosocial Work Stressors on Mental Health Trajectories in Later Life

**DOI:** 10.21203/rs.3.rs-10116154/v1

**Published:** 2026-07-27

**Authors:** Kaori Enatsu-Møll, Marja Aartsen, Tale Hellevik, Ira Malmberg-Heimonen

**Affiliations:** OsloMet – Oslo Metropolitan University

**Keywords:** Longitudinal study, Caregivers, Working conditions, Mental health, Europe, SHARE

## Abstract

**Background:**

In aging societies, concerns about the financial sustainability of pension systems and rising healthcare expenditure have led to increased reliance on informal care and policies aimed at extending working lives. Consequently, a growing number of older adults combine paid work with informal caregiving, which may exacerbate mental health risks, particularly under adverse working conditions. A joint investigation of informal care and working conditions is needed to account for possible effect modification, but this has rarely been done. This study therefore examines how trajectories of informal caregiving and psychosocial work stressors shape mental health trajectories among older adults.

**Methods:**

We used longitudinal data from the Survey of Health, Ageing and Retirement in Europe (SHARE; waves 6, 7, 8, and 9), comprising 13,233 adults aged 50 years and older at baseline from 17 European countries. Cross-domain latent growth models were employed to evaluate the longitudinal associations between informal caregiving, psychosocial work stressors, and mental health.

**Results:**

Results indicate that informal caregivers, as well as those who become caregivers within the household, exhibit poorer mental health trajectories. An increase in efforts or a reduction in rewards at work is also associated with declining mental health. Among women, but not men, the combined effect of informal caregiving and adverse psychosocial working conditions amplifies negative mental health outcomes.

**Conclusions:**

Our findings suggest that an integrated approach to work conditions and care provision may be especially important for protecting women’s mental health. Policy efforts should be coordinated and targeted to improve psychosocial working conditions while supporting informal caregivers.

## Background

In aging societies, increasing longevity and the rising old-age dependency ratio have placed growing pressures on state pensions and healthcare expenditures. Key policy responses aimed at alleviating these pressures include greater reliance on informal care [1] and the prolongation of working lives [2]. As a result, an increasing number of older employees are combining paid work with caregiving responsibilities, which may exacerbate caregiver burden and increase the risk of mental health problems, particularly in the context of stressful working conditions. Understanding how informal caregiving interacts with psychosocial work stressors to influence mental health trajectories in later life has therefore become important for ensuring healthy working caregivers. However, not many empirical studies have investigated caregiving and psychosocial work stressors in tandem. Failing to account for one when examining the other may lead to biased conclusions [3]. The few existing studies that consider them jointly are limited to small samples, cross-sectional designs and single-country contexts [4, 5]. Consequently, it remains unclear how informal caregiving and working conditions together relate to mental health when considering their longitudinal dynamics and policy contexts.

The aim of this study is therefore to provide an integrated perspective on how trajectories of informal caregiving and psychosocial work stressors shape mental health trajectories as people age, using cross-national, longitudinal data from the Survey of Health, Ageing and Retirement in Europe (SHARE). To address potential effect modification arising from cross-national policy heterogeneity, such as long-term care systems and labor-market protection indices, we will incorporate these policy contexts in our analytical models. Examining informal care and work stressors within an integrative framework in a longitudinal setting, enables us to assess how caregiving and work stress are interrelated with mental health trajectories and to identify where policy efforts should be directed. To this end, we examine the following research question: *To what extent are the distinct and combined trajectories of informal caregiving and psychosocial work stressors, associated with mental health trajectories?*

## Informal care and mental health

Informal caregiving is often described as a demanding role that can negatively affect caregivers’ mental health [6-9]. According to the Informal Caregiving Integrative Model [10], adverse health effects arise from caregiving demands and caregivers’ perceived burden and stress. Evidence suggests that people providing care within the same household experience a higher burden compared to non-co-resident carers, as they tend to provide more intensive care and have fewer opportunities to avoid the caregiving situation [11-13]. The increasing availability of longitudinal data provides new insights into the dynamic relationship between caregiving and health [14-16]. These studies found that transitions into caregiving were associated with a decline in mental health. This effect was more pronounced for caregiving inside the household than for caregiving outside the household [11, 12, 15]. Transitions out of caregiving were found to improve mental health in some [17, 18], but not in other studies [14, 19], possibly due to differences in caregiving contexts.

### Psychosocial work stressors and mental health

Psychosocial work stressors are well-established risk factors for the mental health problems of workers [20-22]. The main explanation builds on two influential theoretical models: the job demand–control model [23] and the effort-reward imbalance model [24]. The job demand-control model posits that jobs characterized by high psychological demands in combination with low levels of control constitute “high-strain” jobs that increase the risk of stress-related health problems [23]. Later, to complement this perspective, Siegrist (1996) introduced the effort–reward imbalance model, emphasizing that work stress arises when the efforts invested are perceived to outweigh the rewards workers receive. A recent meta-analysis supports these theories, concluding that both high-strain jobs and greater effort–reward imbalance are significantly associated with poorer mental health [21]. However, the majority of studies measure psychosocial working conditions only at baseline or using cross-sectional data, and few studies have examined trajectories of psychosocial work stressors and mental health development [21, 22]. Recent studies from Sweden examined trajectories of job demands and control [25], and effort-reward imbalance [26], and how these trajectories are associated with mental health. Both studies found that disadvantaged trajectories were associated with poorer mental health. However, these studies did not account for trajectories of mental health in these associations. Thus, it remains unclear whether and to what extent, changes in psychosocial work stressors are associated with changes in mental health over time.

## Work, informal care and mental health

Combining the dual roles of paid work and informal caregiving can be challenging, as competing work and family demands often create time pressure and psychological stress [3, 27, 28]. Drawing on role theory [29, 30] and the job demands–resources framework [31], Clancy et al. (2020) suggest in their conceptual model of eldercare and work, that working caregivers are particularly vulnerable to stress if job demands are coupled with caregiving demands [3]. Although the mental health of working caregivers has been extensively studied, research that integrates psychosocial working conditions into these analyses remains limited. A few studies examining these associations found that caregivers exposed to high job demands are more likely to experience depressive symptoms than those with lower job demands [4, 5]. However, these studies are limited by small, cross-sectional designs and single-country-contexts.

The impact of work and informal care can also vary by gender and across national contexts [32, 33]. Women are more likely than men to take care responsibilities [34], and men and women may manage multiple role of paid work and family care differently [35]. In addition, cross-national policy variation in support for work and care are likely to influence these dynamics. In generous welfare states, long-term care policies such as respite care and pension credits help reduce caregiver burden [36], while labor market policies including sickness benefit can buffer the health impacts of adverse work environment [37]. By contrast, limited support in less generous welfare states is often linked to greater caregiver burden, making it more difficult to balance work and family responsibilities [38]. Yet, prior research has also shown persistent adverse mental health effects among caregivers, independent of welfare regime or policy contexts [16, 33]. Given this, based on the model of eldercare and work by Clancy et al. [3], we expect that, for both men and women and independent of cross-national policy contexts, the negative associations of informal care and work stress with mental health trajectories will be stronger for individuals who experience higher work stress and caregiving simultaneously, compared to caregivers with lower work stress and workers without caregiving responsibilities.

## Hypotheses

Our expectations regarding the associations between trajectories of informal caregiving and psychosocial work stressors, and trajectories of mental health are as follows:

### H1

People with informal caregiving responsibilities, especially caregivers within the same household, have poorer mental health trajectories than people without caregiving responsibilities.

### H2

Workers exposed to higher and/or increasing levels of work stress experience poorer mental health trajectories than workers exposed to lower and/or decreasing levels of work stress.

### H3

The negative associations between trajectories of informal caregiving and work stress, and trajectories of mental health are stronger among people who simultaneously experience higher levels of work stress and caregiving, compared with caregivers experiencing lower work stress and workers without caregiving responsibilities.

## Method

### Data and study population

The data used in this study is derived from the Survey of Health, Ageing and Retirement in Europe (SHARE, release 9.0.0). SHARE is a cross-national and longitudinal study that collects detailed information on health, socioeconomics and social networks among community-dwelling individuals aged 50 and older in Europe and their partners [39]. SHARE began in 2004 in eleven European countries (plus Israel) with data collected at two-year intervals, and 17 additional countries have joined SHARE since the study’s onset. To increase the sample size and ensure the representativeness of the population in the respective countries, the SHARE survey includes refreshment samples throughout the survey.

For this paper, we analyze data from Wave 6,7, 8, and 9, collected between 2014 and 2022, as these waves represent the most recent SHARE data collections, and include the largest number of participating countries. A total of 18 out of 28 countries participated in all four waves: Austria, Belgium, Croatia, Czech, Denmark, Estonia, France, Germany, Greece, Italy, Israel, Luxembourg, Poland, Portugal, Slovenia, Spain, Sweden, Switzerland. Wave 6 individual response rates varied from 28.4% for France to 57.0% for Greece [40]. For more detailed information about SHARE and its methodology, see Börsch-Supan et al. [39]. Throughout the methods and results, Wave 6, 7, 8, and 9 will be referred to as Time 1 (T1), Time 2 (T2), Time 3 (T3), Time (T4) respectively.

**Figure 1:**
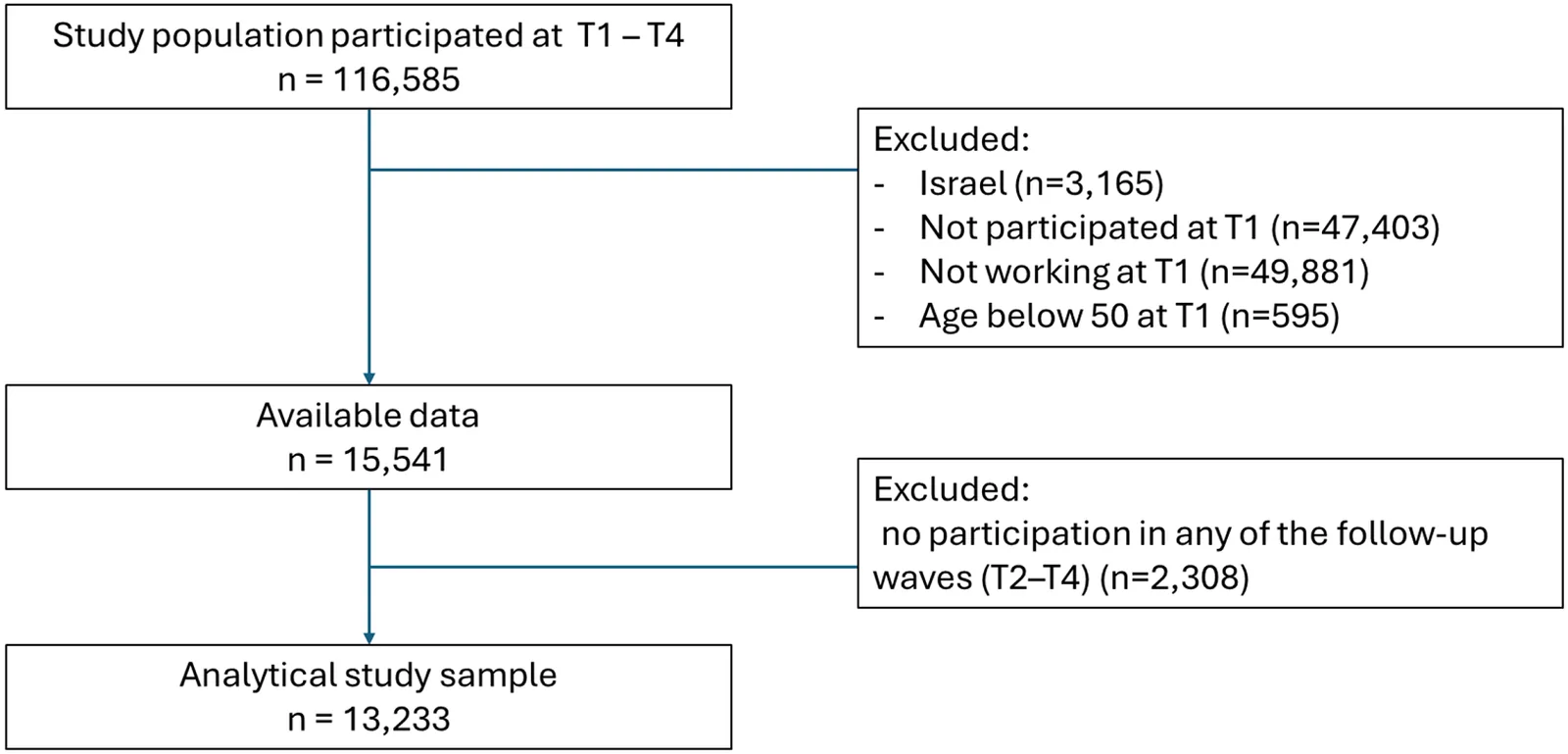
Flowchart of the analytical sample

We applied several data restrictions for this study (see Fig. 1). We excluded Israel because the relevant macro-level policy indicators used in this study were not available for Israel (3,165 respondents). Individuals who did not participate at T1 (i.e., Wave 6) were excluded (47,403 respondents). Individuals not working at T1 were also excluded (49,881 respondents). We further excluded individuals aged < 50 years at T1 (595 respondents). In addition, we excluded those who did not participate in any of the subsequent waves (T2-T4) (2,308 respondents), leaving the final sample of 13,233 respondents.

### Measures

#### Depressive symptoms

Depressive symptoms were used as a proxy for mental health problems. They were assessed with the EURO-D depression scale [41] at each wave, including 12 binary items measuring the presence of the following depressive symptoms over the past month: “depressed mood,” “pessimism,” “suicidality,” “guilt,” “ sleep quality,” “interest,” “ irritability,” “ appetite,” “ fatigue,” “concentration,” “enjoyment,” and “tearfulness.” When adding up the number of symptoms, the scale ranges from 0 to 12 (Cronbach’s alpha = 0.65).

#### Informal care

At each wave, respondents were asked whether they had provided practical help as well as personal care, within and outside the household. For care provided within the household, respondents were asked: “Is there someone living in this household whom you have helped regularly during the last twelve months with personal care, such as washing, getting out of bed, or dressing?” To avoid capturing short-term help during short-term sickness, “regularly” was defined as daily or almost daily. These questions were referred to any person in the household and were asked only if respondents were living in multi-person households. For informal care provided outside the household, respondents were asked: “In the last twelve months, have you personally given any kind of help to a family member outside the household, a friend, or neighbor?” Response categories captured the intensity of help (“almost daily”, “almost every week”, “almost every month”, or “less often”). We restricted informal care outside the household to cases where help was given almost daily, to focus on high-intensity caregivers who are more likely to be at risk of negative health outcomes [7]. Based on these questions, we constructed a binary indicator of caregiving (yes/no) for each wave. To capture changes in caregiving roles over time, we identified four caregiving patterns: “remained non-caregiver”, “started caregiving”, “stopped caregiving”, and “remained caregiver”. These patterns are based on changes in caregiving status between T1 and the last available follow-up wave (T2, T3, or T4) in which the respondent’s caregiving status was measured.

#### Psychosocial work stressors: job control and effort-reward imbalance

The measurement of the work stressors is based on job demand-control [23] and effort-reward imbalance model [24]. For the demand–control model, measurement was restricted to the control dimension as core aspects of job demands are captured by the effort component of the effort-reward imbalance model, reducing multicollinearity. Consistent with previous studies using SHARE [42, 43], *Job control* was assessed using the sum score of two Likert-scale items— “I have very little freedom to decide how I do my work” and “I have an opportunity to develop new skills”—resulting in a scale ranging from 2 to 8, with higher scores indicating lower levels of control at work.

*Effort–reward imbalance* was measured using two items capturing effort and five items capturing reward. The effort items were: “My job is physically demanding” and “I am under constant time pressure due to a heavy workload.” Reward was assessed using five items: “I receive adequate support in difficult situations,” “I receive the recognition I deserve for my work,” “Considering all my efforts and achievements, my salary is adequate,” “My job promotion prospects are poor,” and “My job security is poor.” In line with a previous study by J Siegrist, T Lunau, M Wahrendorf and N Dragano [44], the effort–reward imbalance score was calculated by dividing the sum of the effort items (numerator) by the sum of the reward items—adjusted for the number of items (denominator). The resulting score ranges from 0.25 to 4, with higher values indicating greater work stress. Because our primary interest is for situations where effort exceeds reward, values below 1 were recoded to 1, yielding a final continuous measure ranging from 1 to 4.

#### Policy indicators: sickness benefit provision and caregiver support policy

To account for the potential impact of national policies [45], we control for sickness benefit provision and caregiver support policy. The sickness benefit provision index was measured with a so-called social expenditure approach [46]. Using data from Eurostat [47], we constructed the sickness benefit provision index based on expenditure on cash benefits for sickness and health care, expressed in purchasing power standards (PPS) per inhabitant to ensure comparability across countries, independent of differences in price levels or currency. Because sickness benefits are provided only to employed individuals, the raw expenditure partly reflects each country’s employment rate. To adjust for this, we divided the expenditure by the employment rate in each country in line with van der Wel et al. [48], yielding an index more comparable across welfare systems.

The caregiver support index was based on the index described by Verbakel et al. [49]. It captures long-term care policies in a country aimed at supporting care provided through and within the family [16]. It includes seven types of carer support (counseling, information, respite care, training, financial allowance, pension credits, and care leave). This index ranges from 0 to 1, with higher values representing stronger support (See details Verbakel et al. [49]). Country details of the two policy indicators are summarized in Table 1.

#### Socio-demographic variables: age, education, income, living alone, gender

Several socio-demographic variables were included. Age (in years) was controlled for, given its potential association with informal caregiving and mental health [9]. With respect to socioeconomic factors, people with higher education and higher income may have better health, resources for informal caregiving [50] and better working conditions [51]. In SHARE, education was measured according to the International Standard Classification of Educational Degrees (ISCED-97) with the following seven categories: (0) None, (1) Primary level of education, (2) Lower secondary level of education, (3) Upper secondary level of education, (4) Postsecondary non-tertiary education, (5) First stage of tertiary education (6) Second stage of tertiary education. Income position was measured using household income percentiles (1–10), where higher percentile values indicate higher household income. We also controlled for living alone, based on respondents’ household size, because caregiving within the household does not occur among individuals living alone. In addition, we estimate the models separately for men and women to take into account potential gender differences in the relationships between informal care, work, and mental health [3, 45, 52]. All socio-demographic variables were taken from baseline (T1).

#### Analytical strategies

All analyses were conducted with Stata 19.5 and R version 4.5.2. To examine trajectories of mental health and psychosocial work stressors, we estimated latent growth models (LGMs) [53, 54]. A LGM is a type of structural equation model that estimates the average initial level (intercept) and average rate of change (slope) over time, as well as the variances in these parameters. First, univariate linear latent growth models (LGMs) were fitted to examine trajectories of depressive symptoms, job control, and effort–reward imbalance. These models estimated intercepts and slopes for each construct and demonstrated adequate model fit (results not shown). Because informal caregiving was measured as a binary variable, LGMs were not estimated for caregiving. Instead, observed changes in caregiving status over time were considered as trajectories of informal caregiving.

**Figure 2:**
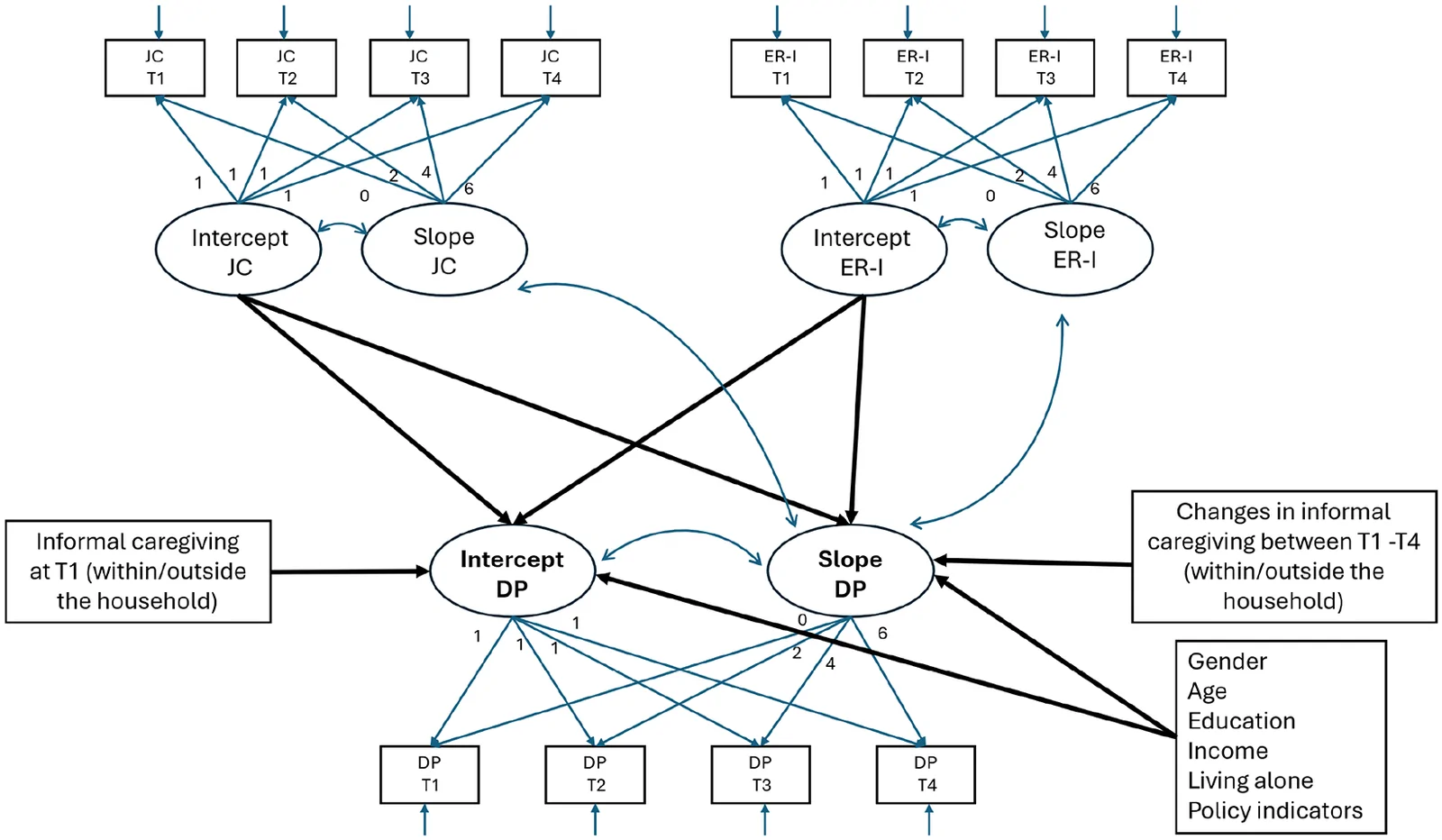
Analytical Model DP = Depressive symptoms, JC= Job control, ER-I= Effort-Reward Imbalance. Single-headed arrows represent regression effects. Double-headed arrows represent correlations.

After establishing the linearity of the growth trajectories and checking for satisfactory fit in the univariate LGMs, we conducted cross-domain LGMs to examine associations between the growth parameters intercept and slope of each of the trajectories. The cross-domain LGMs included six sets of regression equations and five sets of correlations (see Fig. 2). In the regression part, the intercept of depressive symptoms was regressed on informal caregiving at T1 (within and outside the household respectively), and the intercepts of psychosocial work stressors (job control and effort–reward imbalance). To take into account variations in cross national contexts and individual factors, this regression was also adjusted for macro-level policy indicators and individual sociodemographic characteristics. The slope of depressive symptoms was regressed on changes in caregiving status over time, the intercepts of psychosocial work stressors. Similar to the intercept of depressive symptoms, this regression was also controlled for macro-level policy indicators and sociodemographic covariates. To examine the interrelation between informal caregiving and changes in work stress over time, the slopes of job control and effort–reward imbalance were additionally regressed on informal caregiving status at T1 (within and outside the household respectively). Although reversed causality is possible, that is, psychosocial work stressors at baseline influence subsequent changes in caregiving roles, we assume that caregiving is primarily determined by the needs of the care recipient. Thus, we focused on the direct effect of caregiving status on the slopes of psychosocial work stressors, and not vice versa. In the correlation part, correlations between the intercept and slope of each construct estimated in the LGMs (depressive symptoms, job control, and effort–reward imbalance), as well as correlations between the slope of depressive symptoms and the slopes of job control and effort–reward imbalance, were included. Missing data were handled using Full Information Maximum Likelihood (FIML). All continuous covariates were mean-centered. Model fit was evaluated using the root mean square error of approximation (RMSEA), Tucker–Lewis Index (TLI), and comparative fit index (CFI). Model fit was considered good when CFI and TLI were ≥ 0.95 and RMSEA was ≤ 0.05 [55]. All analyses were conducted separately for men and women.

## Results

### Descriptive statistics

Table 2 presents the descriptive statistics for the study sample. Gender differences were observed for all study variables. Average depressive symptoms at T1 were higher among women (M = 2.30, SD = 2.05) than men (M = 1.60, SD = 1.73). A larger proportion of women provided informal caregiving both inside and outside the household at T1 compared with men (inside household: 6.32% vs. 4.80%; outside household: 6.41% vs. 3.32%). The average age of the analytical sample was 58.71 years for men (SD = 4.92) and 57.59 years for women (SD = 4.62). The average level of education was slightly higher among women (M = 3.54, SD = 1.31) than men (M = 3.41, SD = 1.35). In contrast, the mean income position percentile was higher for men (M = 7.01, SD = 2.84) than for women (M = 6.75, SD = 2.85).

### Latent growth analyses

Table 3 presents the results from the LGMs for men and women. Both models had an excellent fit (χ^2^ =709.248, df = 122, RMSEA = 0.036, CFI = 0.980, TLI = 0.974; χ^2^ =766.548, df = 122, RMSEA = 0.038, CFI = 0.979, TLI = 0.972). The intercepts of depressive symptoms represent the average level of depressive symptoms when all covariates are set to zero. The slopes of depressive symptoms were positive for both men and women, and reflected a yearly increase in depressive symptoms (B_men_=0.090, B_women_=0.161).

### Informal care

The effect of caregiving within the household at T1 on the intercept of depressive symptoms was significant for both men and women (B_men_=0.236, B_women_=0.414), suggesting that being an informal caregiver within the household was associated with more depressive symptoms. With respect to the effects of informal caregiving on the slope of depressive symptoms, transitions into caregiving within the household were related to a faster increase in depressive symptoms over an eight-year period than those remaining non-caregivers, for both genders (B_men_=0.058, B_women_=0.099). For women, we also observed significant direct effects of caregiving outside the household on the slope of depressive symptoms. Transitioning into caregiving and remaining in a caregiving role were both associated with a faster increase in depressive symptoms, whereas transitioning out of caregiving was associated with a slower increase in depressive symptoms compared to those who remained non-caregivers.

### Psychosocial work stressors

The direct effect of the intercept of effort-reward imbalance on the intercept of depressive symptoms, was observed for both men and women (B_men_=1.473, B_women_=1.945). Correlations between the slopes indicate that changes in the work stressors are associated with changes in depressive symptoms. Specifically, an increase in effort relative to the rewards of the job was significantly related to a faster increase in depressive symptoms for both genders (r_men_=0.391, r_women_=0.369). For women, an increase in low job control was also linked to a faster increase in depressive symptoms (r = 0.490).

### The interplay between informal care and psychosocial work stressors

We argued that caregiving and work stress are not independent of each other. For women, caregiving within the household at baseline were significantly associated with an increase in effort-reward imbalance (B = 0.010), which is associated with a greater rise in depressive symptoms (r = 0.369). This is partly in line with our expectation (H3), suggesting that women exposed to higher levels of work stress combined with caregiving responsibilities experience a greater increase in depressive symptoms than caregivers with lower work stress and women without caregiving responsibilities. In men, however, caregiving was not associated with an increase in psychosocial work stressors, suggesting that the effects of these factors on mental health are additive rather than interactive.

### Sensitivity analyses

We conducted several sensitivity tests (Appendix S1-S3). First, the analyses were repeated excluding Wave 7, as this wave had a substantially smaller sample size for the relevant variables and could therefore reduce the reliability of the parameter estimates in the growth model. Second, the analyses were repeated for a subsample restricted to individuals working full-time at T1, to account for potential differences in health effects between full-time and part-time workers [3], such as time constraints and the additional burden of combining full-time employment with informal caregiving. Lastly, we conducted an additional sensitivity analysis excluding individuals who reported being retired at any follow-ups to account for the potential influence of retirement on changes in mental health [56]. Overall, due to the differences in sample size, some associations lost significance in the models with the smaller sample sizes, but effects were generally in the same direction and therefore did not substantially alter our conclusions. There were, however, a few exceptions. Compared with the main analysis for men (Table 3), the direct effect of intercept job control on intercept depressive symptoms became significant in the model including Waves 6, 8, and 9 (Appendix S1). This may reflect a suppression effect in the main analysis, that is, the measurement variability associated with the sparse Wave 7 data attenuated the effect in the full model, although this requires further investigation. Among women in the full-time employment sample (Appendix S2), remaining a caregiver within the household became significantly associated with a faster increase in depressive symptoms compared with remaining a non-caregiver. This may be due to the additional burden and time constraints of combining full-time work with informal caregiving.

## Discussion

### Main outcomes

In the present study, we examined the distinct and combined longitudinal associations between trajectories of informal caregiving and psychosocial work stressors, and trajectories of mental health, while taking into account individual sociodemographic characteristics and country-level policy indicators. Our first hypothesis, that people with informal caregiving responsibilities have poorer mental health trajectories compared to people without caregiving responsibilities, is partly supported by the data. In line with our expectations, we observed that both men and women with caregiving responsibilities at baseline, as well as those transitioning into caregiving within the household, experienced greater and faster increases in depressive symptoms. Among women, entering or remaining in caregiving roles outside the household was also associated with increased depressive symptoms. With respect to the second hypothesis, our study reveals that workers exposed to higher and increasing imbalance in efforts and rewards in the job were related to poorer mental health trajectories, supporting the effort-reward imbalance model. Furthermore, a significant association between initial status of care provision within the household and the development of effort–reward imbalance was observed among women. This provides some support for the synergistic burden experienced by those combining caregiving responsibilities with paid work under psychosocial work stress (H3).

### The unique effects of informal care and psychosocial work stressors

Overall, our findings are in line with previous studies documenting poorer mental health among working men and women who provide informal care [7, 9, 57]. For both men and women, the initial status of caregiving and the transition into caregiving had a significant negative mental health impact when the care was provided within the household, independent of psychosocial working conditions. Caregiving outside the household, however, was less consistently linked to poor mental health than caregiving within the household, with faster increases in depressive symptoms observed only among women who entered and remained in caregiving roles. A possible explanation is that within-household care activities tend to be longer and more intense care than caregiving outside the household, as well as limited opportunities to avoid and recover from caregiving burdens, leading to a greater risk of mental health problems [58, 59]. In contrast, caregiving for non-residential recipients may involve different and less stressful tasks and can even be experienced as rewarding, for example by providing a sense of fulfillment or meaning [60]. Moreover, caregiving outside the household may reflect a selection process in which generally healthier individuals are more likely to take on these roles [15]. There is a need for further research examining heterogeneity in caregiving experiences by care location, as well as potential benefits of caregiving.

Our findings also highlight the importance of psychosocial work stressors, as changes in effort–reward imbalance were positively associated with changes in depressive symptoms, independent of baseline depressive symptoms, psychosocial work stressors, caregiving status, sociodemographic factors, and macro-level national policy indicators. This finding is consistent with previous studies [21, 22], providing support for a longitudinal relationship between psychosocial work stress and poor mental health. In contrast with our expectations, however, we also observed that higher initial effort–reward imbalance was linked to a slower increase in depressive symptoms. It is unclear why high initial work stressors do not accelerate the increase in depressive symptoms over time. We can only speculate about explanations, but one can be that individuals who perceive high stress at work change jobs, adjust workloads, or engage in activities outside work that buffer or alleviate the rise in depressive symptoms [61-64]. Further investigation is necessary to support this.

### The longitudinal association between informal caregiving and psychosocial work stressors

With regard to the longitudinal relationship between informal caregiving and psychosocial work stressors, we identified some evidence of their interlinked nature. In particular, informal caregiving was significantly associated with an increased perception of effort–reward imbalance at work among women, but not among men, suggesting that women may be more vulnerable to the cumulative pressures of combining paid work and caregiving. One possible explanation for this gender difference is that women often spend more time on caregiving and are more likely to provide more demanding care, which may increase time pressure and stress [33]. It may also partly reflect gendered role expectations. Women’s socially embedded role as primary caregivers often entails greater care responsibility and more emotionally intensive care, which may make caregiving stress spill over more easily into the work domain [65]. In contrast, men’s stronger orientation toward paid work and task-oriented masculine norms may place work and caregiving as more detached roles [66], which may weaken the link between caregiving stress and work-related stress. It could also be that male caregivers more often have greater financial resources, allowing them to outsource some care or household tasks and reduce their overall burden [67]. Further research is needed to clarify the mechanisms underlying these gender differences in working caregivers’ health.

### Limitations and strengths

This study has several limitations. First, we did not differentiate between full-time and part-time work, as our primary focus was the psychosocial working conditions of workers in general. While additional sensitivity analyses restricted to full-time workers yielded largely similar results, our findings also suggest that full-time working women may experience an additional burden from combining work and caregiving responsibilities (Appendix Table S2). Therefore, further analysis may be needed to account for working arrangements and to examine their potential effects. Second, working status was measured only at baseline, and psychosocial work stress was modeled as continuing to develop over the study period to avoid too complex models. Even though restricting the analyses to individuals who remained workers did not alter the conclusions (Appendix Table S3), there may be work trajectories which were not captured, such as job transitions or changes in employment conditions. Third, the causal direction between trajectories of psychosocial work stress and depressive symptoms were not determined, as this was beyond the scope of the present study. Nonetheless, the findings provide longitudinal evidence of their associations, indicating the need for future research to examine temporal ordering and potential causal pathways. Fourth, linear effects were assumed for model simplicity and interpretability. While overall model fit was adequate, non-linear trajectories were not captured, potentially obscuring more nuanced patterns of change associated with life transitions, such as the loss of a spouse or retirement. Future studies could extend this work by applying models that allow for non-linear change, enabling examination of trajectories that account for key life-course transitions. Finally, although relevant country-specific policy indicators were included to account for cross-national differences [16, 48, 49], other structural or cultural factors not captured in our analysis may have modified the observed associations. Nonetheless, the use of country-specific policy indicators, instead of welfare-state typologies, avoided obscuring the country differences in labor market and care policies [16, 37]. Furthermore, the relatively small number of countries included in the present study (N = 17) limited the feasibility of multilevel modelling, as country-level random-effects estimates may be biased or unreliable when based on a limited number of higher-level clusters [68]. Future research could further develop this study through the application of multilevel or moderation approaches to assess how national and policy contexts influence the associations between work, caregiving, and mental health.

Despite these limitations, this study has some important strengths. The data includes a large cross-national sample with over eight years of follow-up, enabling examination of trajectories of informal caregiving, psychosocial work stress, and depressive symptoms in later life. To our knowledge, this is one of the first studies to examine trajectories of psychosocial working conditions and informal caregiving in relation to mental health trajectories. Using latent growth modeling, we simultaneously modeled trajectories of psychosocial work stress, caregiving patterns, and their cross-sectional and longitudinal associations with depressive symptoms, providing a more nuanced understanding of the interplay between work, care, and mental health in later life.

## Conclusion

Given the growing policy emphasis on extending working lives while simultaneously relying on families to provide informal care, our findings highlight an important tension. Efforts to prolong employment may conflict with the increasing need for informal care, as care provision can exacerbate mental stress among workers and potentially undermine the ability to remain in the labor market, especially under adverse working conditions. Effective policy responses therefore require coordinated efforts among key stakeholders such as policymakers, employers, and professional care providers tailored to specific contexts and needs. Such efforts may include financial and organizational support, for example through paid care leave, flexible working-time arrangements, and access to caregiver support services. Creating structures that enable workers to balance paid work and informal caregiving is essential both for protecting their mental health and for sustaining longer working lives in the context of growing care needs.

Our results also highlight the need for integrated research frameworks that examine work stressors and care provision in tandem, rather than as separate domains, to better understand mental health development in later life. Treating these factors in isolation may limit insights into how their combined effects shape mental health among working caregivers, particularly in the context of ongoing demographic change. Future research would benefit from longitudinal designs that capture the dynamic and cumulative interplay between trajectories of work stress, caregiving responsibilities, and mental health. In addition, theoretical models could benefit from incorporating gender perspectives and macro-level factors, as these may shape how individuals experience and manage work and caregiving demands. Further research focusing on the roles of these factors is warranted.

## Supplementary Material

This is a list of supplementary files associated with this preprint. Click to download.

• Appendix.docx

## Figures and Tables

**Table 1.** Policy indicators by country

Country	N	Sickness benefit provision	Caregiver support
Austria	403	33.88	0.86
Belgium	1,251	39.27	0.43
Switzerland	806	38.29	-
Germany	1,188	41.36	0.86
Denmark	1,337	29.79	1.00
Spain	808	25.06	0.71
France	713	37.57	0.57
Greece	854	16.59	0.57
Italy	960	28.38	0.57
Sweden	870	30.42	0.71
Czech	698	19.98	0.43
Poland	292	13.11	0.00
Luxembourg	288	49.68	0.86
Slovenia	561	23.12	0.14
Estonia	1,607	12.06	-
Croatia	388	18.29	-
Portugal	209	18.25	0.43
Total	13,233		

**Table 2.** Descriptive statistics of the study sample

	Men (n = 6,334)				Women (n = 6,899)				
	N	%	M	SD	N	%	M	SD	diff.
Depressive symptoms (0–12)									
T1	6,103		1.60	1.73	6,818		2.30	2.05	***
T2	874		1.31	1.55	930		2.08	2.10	***
T3	3,660		1.55	1.70	4,051		2.15	1.95	***
T4	4,431		1.64	1.78	5,010		2.30	2.10	***
Care within the household at T1									
No	5,379	95.20			5,452	93.68			***
Yes	271	4.80			368	6.32			***
Caregiving patterns: within the household									
Remained non-caregivers	5,258	92.20			5,304	89.70			***
Started caregiving	173	3.02			239	4.04			***
Stopped caregiving	182	3.18			212	3.59			***
Remained caregivers	92	1.61			158	2.67			***
Care outside the household at T1									
No	6,117	96.68			6,453	93.59			***
Yes	210	3.32			442	6.41			***
Caregiving patterns: outside the household									
Remained non-caregivers	5,961	94.14			6,141	89.01			***
Started caregiving	161	2.54			316	4.58			***
Stopped caregiving	166	2.62			330	4.78			***
Remained caregivers	44	0.69			112	1.62			***
Job control (2–8)									
T1	4,708		4.16	1.39	5,427		4.29	1.43	***
T2	549		4.21	1.38	579		4.40	1.40	**
T3	2,065		4.12	1.38	2,348		4.24	1.42	***
T4	1,878		4.08	1.38	2,149		4.30	1.41	***
Effort-reward imbalance (1–4)									
T1	4,591		1.14	0.29	5,291		1.15	0.32	**
T2	530		1.15	0.28	560		1.15	0.31	
T3	1,990		1.12	0.27	2,277		1.13	0.30	
T4	1,803		1.11	0.26	2,073		1.12	0.26	
Living alone									
No	5,630	88.89			5,780	83.78			***
Yes	704	11.11			1,119	16.22			***
Age	6,334		58.71	4.92	6,899		57.59	4.62	***
Education	6,290		3.41	1.35	6,824		3.54	1.31	***
Income position (percentile)	6,334		7.01	2.84	6,899		6.75	2.85	***
Sick benefit provision	6,334		28.15	10.07	6,899		28.20	10.38	
Caregiver support policy index	5,080		0.63	0.25	5,352		0.63	0.25	

Note. N=number of observations; M= mean; SD= standard deviation; At T2 (Wave 7 survey), the survey primarily consists of retrospective questionnaires and has a small sample size for the regular panel survey.

**Table 3.** Latent Growth Models for men and women

		Men			Women		
		Est	SE	sig.	Est	SE	sig.
Intercept Depressive Symptoms		−0.461	0.192	**	−0.300	0.227	
Slope Depressive Symptoms		0.090	0.039	**	0.161	0.044	***
Intercept Job Control		4.202	0.023	***	4.349	0.023	***
Slope Job Control		−0.011	0.006		−0.003	0.006	
Intercept Effort-Reward Imbalance		1.145	0.005	***	1.156	0.005	***
Slope Effort-Reward Imbalance		−0.005	0.001	***	−0.003	0.001	***
Correlations							
Intercept Depressive Symptoms ↔ Slope Depressive symptoms		−0.200	0.021		−0.278	0.028	
Intercept Job Control ↔ Slope Job Control		−0.386	0.021	***	−0.340	0.019	
Intercept Effort-Reward Imbalance ↔ Slope Effort-Reward Imbalance		−0.676	0.001	***	−0.315	0.001	***
Slope Job Control ↔ Slope Depressive symptoms		0.067	0.006		0.490	0.002	**
Slope Effort-Reward Imbalance ↔ Slope Depressive symptoms		0.391	0.000	***	0.369	0.000	***
Covariates → Intercept Depressive Symptoms							
Care within the household at T1 (yes = 1)		0.236	0.115	**	0.414	0.123	***
Care outside the household at T1 (yes = 1)		0.064	0.138		0.188	0.117	
Intercept Job Control		0.066	0.042		0.055	0.054	
Intercept Effort-Reward Imbalance		1.473	0.196	***	1.945	0.267	***
Sickness Benefit Provision		0.016	0.003	***	0.025	0.004	***
Caregiver support policy		−0.469	0.111	***	−0.465	0.133	***
Age		−0.010	0.005		−0.008	0.007	
Living alone (yes = 1)		1.226	0.323	***	0.973	0.385	**
Education		−0.009	0.018		−0.066	0.023	***
Income position		−0.023	0.010	**	−0.052	0.012	***
Covariates → Slope Depressive Symptoms							
Care within the household (ref. remained non-caregiver)							
Started caregiving		0.058	0.024	**	0.099	0.024	***
Stopped caregiving		0.005	0.028		−0.001	0.030	
Remained caregivers		0.053	0.044		0.049	0.041	
Care outside the household (ref. remained non-caregiver)							
Started caregiving		0.032	0.026		0.092	0.023	***
Stopped caregiving		0.016	0.031		−0.055	0.026	**
Remained caregivers		−0.062	0.077		0.115	0.050	**
Intercept Job Control		0.004	0.009		−0.012	0.011	
Intercept Effort -Reward Imbalance		−0.100	0.038	***	−0.113	0.049	**
Sickness Benefit Provision		0.002	0.001	***	0.003	0.001	***
Caregiver support policy		0.041	0.024		−0.001	0.028	
Age		0.002	0.001		0.000	0.002	
Living alone (yes = 1)		−0.219	0.073	***	−0.134	0.080	**
Education		−0.007	0.004		−0.002	0.005	
Income		0.003	0.002		0.006	0.002	**
Care within the household at T1 → Slope Job Control		0.017	0.024		−0.013	0.023	
Care within the household at T1 → Slope Effort-Reward Imbalance		0.004	0.004		0.010	0.005	**
Care outside the household at T1 → Slope Job Control		−0.039	0.028		0.018	0.020	
Care outside the household at T1 → Slope Effort-Reward Imbalance		0.006	0.005		−0.002	0.004	
N		4,503			4,533	
Model fit	CFI	0.980			0.981	
	TLI	0.970			0.978	
	RMSEA	0.029			0.029	

Note. Est=coefficients; SE= standard error; N=number of used observations in the analyses; CFI=comparative fit index; TLI=Tucker–Lewis Index; RMSEA= root mean square error of approximation

** p < 0.05

*** p < 0.01

## Abbreviations

SHARE: Survey of Health, Ageing and Retirement in Europe
T1: Wave 6 of SHARE
T2: Wave 7 of SHARE
T3: Wave 8 of SHARE
T4: Wave 9 of SHARE
PPS: Purchasing power standards
ISCED-97: International Standard Classification of Educational Degrees
LGMs: Latent Growth Models
RMSEA: Root mean square error of approximation
TLI: Tucker–Lewis Index
CFI: Comparative fit index
